# Relationship between Semenogelins bound to human sperm and other semen parameters and pregnancy outcomes

**DOI:** 10.1186/s12610-017-0059-6

**Published:** 2017-08-08

**Authors:** Kazumitsu Yamasaki, Kaoru Yoshida, Miki Yoshiike, Kazuhiko Shimada, Hiroyuki Nishiyama, Satoru Takamizawa, Kaoru Yanagida, Teruaki Iwamoto

**Affiliations:** 1 Department of Urology, Tsukuba Gakuen Hospital, Tsukuba, Japan; 2 Faculty of Biomedical Engineering, Toin University of Yokohama, Yokohama, Japan; 3 Department of Urology, St Marianna University School of Medicine, Kawasaki, Japan; 4 Institute for Central Clinic, Shimotsuke, Japan; 5 Department of Urology, Faculty of Medicine, University of Tsukuba, Tsukuba, Japan; 6 Center for IVF and Infertility, International University of Health and Welfare Hospital, Nasushiobara, Japan

**Keywords:** Seminal plasma protein, Male infertility, Sperm motility, Semenogelin, IUI, IVF, ICSI, Pregnancy outcomes

## Abstract

**Background:**

Semenogelins (SEMGs) are major components of human seminal vesicle secretions. Due to SEMG’s sperm-motility inhibitor, a significant negative correlation between sperm motility and the proportion of SEMG-bound spermatozoa (SEMG+) was found in asthenozoospermic patients. SEMGs also show intrinsic inhibitory capability for sperm capacitation; however, studies on actual clinical specimens have not been conducted.

**Methods:**

To reveal the relationship between SEMGs and the fertilizing capacity of sperm from male infertile patients who are not restricted to asthenozoospermia, we measured the proportion of SEMG+ in the spermatozoa of 142 male infertile patients. The pregnancy outcomes in partners of these patients were retrospectively analyzed using questionnaires.

**Results:**

Among examined semen parameters, only the total SEMG-unbound sperm count showed a tendency to be different between the spontaneous pregnancy or intra-uterine-insemination-pregnancy groups and in-vitro-fertilization- or intracytoplasmic-sperm-injection-pregnancy groups. It was elevated in the former group, which includes patients who used in vivo fertilization.

**Conclusions:**

The total SEMG-unbound sperm count would be a relevant parameter for in vivo fertilization. This result suggests that SEMGs inhibit ectopic capacitation before sperm reach the fertilization site and that the number of total SEMG-unbound sperm is a parameter directly linked to the possibility of in vivo fertilization.

**Electronic supplementary material:**

The online version of this article (doi:10.1186/s12610-017-0059-6) contains supplementary material, which is available to authorized users.

## Résumé

**Contexte:**

Les séménogélines (SEMG) sont des composantes principales des sécrétions des vésicules séminales humaines. En raison de la présence sur SEMG d’un inhibiteur de la mobilité des spermatozoïdes, une corrélation significative a été rapportée entre la mobilité des spermatozoïdes et le pourcentage de spermatozoïdes liés à SEMG chez des patients asthénozoospermiques. Les SEMG possèdent aussi une capacité intrinsèque d’inhibition de la capacitation; aucune étude n’a cependant été réalisée sur des échantillons de sperme utilisés en pratique clinique quotidienne.

**Matériel et méthodes:**

De façon à mettre en évidence une relation entre les SEMG et la capacité fécondante de spermatozoïdes d’hommes inféconds qui ne soient pas seulement des patients asthénozoospermiques, nous avons mesuré la proportion de spermatozoïdes SEMG+ (liés à SEMG) chez 142 patients inféconds. L’issue des grossesses chez les partenaires de ces patients a été rétrospectivement analysée à partir de questionnaires.

**Résultats:**

Parmi les paramètres spermatiques analysés, seul le nombre total de spermatozoïdes non liées à SEMG montre une tendance à être différent entre le groupe de grossesses obtenues spontanément ou par insémination intra-utérine et le groupe de grossesses obtenues par fécondation in vitro ou injection intra-cytoplasmique d’un spermatozoïde. Ce nombre total était élevé dans le premier groupe qui incluait des patients utilisant une fécondation in vivo.

**Conclusions:**

Le nombre total de spermatozoïdes non liés à SEMG pourrait être un paramètre pertinent pour la fécondation in vivo. Ces résultats suggèrent que les SEMG inhibent la capacitation ectopique avant que les spermatozoïdes n’atteignent le site de fécondation, et que le nombre total de spermatozoïdes non liés à SEMG est. un paramètre directement lié à la possibilité de fécondation in vivo.

**Mots-clés:**

Protéine plasmatique séminale, Infécondité masculine, Séménogélines, IIU, FIV, ICSI, Issues de grossesses

## Background

Globally, approximately 15% of couples of reproductive-ages do not achieve pregnancy within 1 year of regular unprotected sexual intercourse and seek fertility assessment [1]. Among these couples, eventually 5% remain unwillingly childless. Male infertility-associated factor, together with abnormal semen parameters, contributes to 50% of involuntarily childlessness in couples. Semen analysis is the most widely used biomarker to predict the male fertility potential. Its result gives a wide array of information on the functional status of the whole male sex system, such as hormone axis, seminiferous tubules, epididymis, and accessory sex glands. On the other hand, as it is a complex test, semen analysis should ideally be conducted in andrology laboratories by experienced technicians in the presence of internal and external quality controls; validation of test systems; and quality assurance during all testing processes, some of which are often difficult to implement in practice [2]. These difficulties often prevent comparative studies of multicenter data. Additionally, their ability to predict male fertility potential has been questioned, unless the parameters truly are at extremely low levels. For example, among infertile men, semen analysis results are normal in up to 40% of cases [3].

To overcome these insufficiencies of routine semen analysis for diagnosis, specialized andrology tests have emerged to account for sperm dysfunction. Formerly, particularly before the advent of intracytoplasmic sperm injection (ICSI), tests that assessed antisperm antibodies, sperm hyperactivation, and acrosome reaction; sperm binding; and penetration to the human zona pellucida were used to investigate males with unexplained infertility [4]. These tests hold the capability to reveal problems that may exist in each step of conventional in vitro fertilization (IVF), and help to predict the fertilizing potential of sperm in this procedure. However, treatments for many of these potential issues are often extremely difficult or do not exist. Furthermore, the efficacy of assisted reproductive techniques (ART), particularly ICSI, is essentially unaffected by these problems; thus, clinical usage of these tests is highly restricted. Based on the increased knowledge regarding the molecular mechanisms that regulate sperm function, oxidative stress levels and nuclear DNA integrity in the determination of the functional competence of human spermatozoa have been emphasized. These parameters seem to better correlate with male fertility than does standard semen analysis, and tests to measure them have become clinically available. However, to be used routinely, these tests should meet the following requirements, including standardization of protocols, validation of results in larger trials, and cost-effectiveness analyses.

Semenogelins (SEMGs) are major components of human seminal vesicle secretions and are comprised of two closely-related proteins: SEMG 1 and 2 [5]. These two proteins and their degradation byproducts hold a variety of physiological roles [6]. Ejaculated semen immediately turns into a gelatinous meshwork, in which cross-linking SEMGs are major components and sperms are entrapped. During liquefaction, SEMGs are degraded into low molecular mass proteins by a prostate-specific antigen (PSA), and sperms start to move [7]. However, it was shown that seminal hyperviscosity had no relation to the degree of SEMG degradation [8], thereby indicating that SEMGs do not inhibit sperm motility through seminal viscosity. One of the fragments of the degraded SEMGs, a seminal plasma motility inhibitor (SPMI), inhibits the motility of both demembranated spermatozoa and intact spermatozoa. SEMGs have an inhibitory effect on the acrosome reaction induced by fetal cord serum ultrafiltrate. At present, SEMGs and their degradation peptides are considered decapacitation factors that prevent human sperm capacitation. The mechanism underlying these physiological roles of SEMGs and their degradation fragments are still unclear. Interestingly, our group recently reported the association between SEMGs and spermatozoa in infertile men with asthenozoospermia [9].

Based on the clinical relevance of SEMGs to male factor infertility and their putative physiologic role, we hypothesized that SEMGs might be targets for developing new sperm function tests that can guide couples with male factor infertility to select the most appropriate treatment. In this study, we examined the condition of SEMGs bound to sperm from infertile patients and retrospectively analyzed the pregnancy outcomes of these patients’ partners using questionnaires. These results can provide information on the new sperm function tests using SEMGs by indicating whether they are practical for the evaluation of sperm function beyond motility.

## Methods

### Subjects

One hundred and forty-two cases of male infertile patients who had visited the reproduction center of the International University of Health and Welfare Hospital (IUHW) from August 2012 to March 2013 were included in this study. All infertile couples had undergone treatments at IUHW. The policy of infertility treatment at IUHW, according to the semen condition of the male partner, is as follows: for normal findings or in the case of mild oligozoospermia and /or asthenozoospermia, natural pregnancy or intra-uterine insemination (IUI) using the timing method are considered as the first choice, followed by a “step-up” to IVF/ICSI when pregnancy is not achieved; for severe oligozoospermia and/or asthenozoospermia, IVF/ICSI is conducted from the beginning. Furthermore, partners of patients with psychogenic erectile dysfunction or intravaginal ejaculation disorder are initially treated with IUI.

As part of this study, a physical examination and evaluation of male infertility was performed for each subject. During the period of follow-up until the end of 2014, we sent questionnaires to all male infertile patients to obtain data of pregnancy outcomes and collected the answers from 96 of them, which were accompanied by semen analysis data and SEMG+ measurements taken at the first medical examination. Of these 96 partners of patients, 36 were confirmed to achieve pregnancy by any means of IUI, IVF, ICSI or spontaneous conception (“Pregnancy group”), while remaining 60 partners of patients kept unpregnant by the end of the follow-up period (“Non-pregnancy group”). Thirteen normal healthy male subjects whose wives were in the twentieth week or later of spontaneous pregnancy and who received prenatal examinations in the hospital were recruited to participate as “control” subjects in this study after it was confirmed that they did not have any types of intrascrotum abnormality, including varicocele. Informed consent was obtained from all participants for the use of the data obtained from a physical examination, semen, and blood samples. The study protocol was approved by the Institutional Review Board at IUHW and at Toin University of Yokohama in Japan.

### Semen analysis and hormone measurements

Semen samples were obtained via masturbation following sexual abstinence for at least 48 h. Specimens were allowed to liquefy for up to 1 h after ejaculation at room temperature. Complete liquefaction was certified macroscopically according to the WHO manual by confirming that the semen becomes homogeneous and quite watery, and only small areas of coagulation remain. Manual semen analysis was performed according to the WHO manual to determine semen volume and sperm concentration. For analysis of sperm motility, the SMAS™ (Ditect, Tokyo, Japan) system was used. Additionally, the serum levels of luteinizing hormone (LH), follicle-stimulating hormone (FSH), and testosterone (T) were measured using a chemiluminescent immunoassay (SRL Inc., Tokyo, Japan). The reference ranges for LH, FSH, and T were 0.79–5.72 mIU/ml, 2.00–8.30 mIU/ml, and 1.31–8.71 ng/ml, respectively. The intra-assay coefficient of variation (CV) for LH, FSH, and T were 3.03%, 3.74%, and 5.13%, respectively. The inter-assay CV for LH, FSH, and T were 1.84%, 0.43%, and 3.99%, respectively.

### Indirect immunofluorescence assay

Following semen analysis, semen samples were refrigerated until use. Pretreatment and staining methods were the same as described in the previous study [9]. Briefly, semen samples were layered onto 65% Percoll/HEPES-buffered saline (HBS; 130 mM NaCl, 4 mM KCl, 1 mM, CaCl_2_, 0.5 mM MgCl_2_, 14 mM fructose and 10 mM HEPES, pH 8.0) and centrifuged at 1200 g for 30 min at 25 °C. The resulting pellet was fixed with 2% paraformaldehyde phosphate buffer, pH 7.4. The fixed sperm were then washed twice with HBS, mixed with 25% block-Ace (Dainippon Sumitomo Pharma, Osaka, Japan) and then incubated at 37 °C for 1 h, then washed with HBS at 3300 g for 10 min at 25 °C. An aliquot of the washed sperm was incubated with 1 μg/mL anti-seminal plasma motility inhibitor (SPMI) mouse immunoglobulin G (IgG) that recognized a part of the SPMI region (138–154: GTQNPSQDSGNSPSGKG) of SEMG (monoclonal antibody, previously described as anti-Sg antibody F11) or mouse IgG isotype control (DAKO Japan, Tokyo, Japan) at 37 °C for 1 h [10]. Sperm were then washed twice with HBS at 3300 g for 10 min at 25 °C. Washed sperm were incubated with Alexa 488-conjugated anti-mouse IgG (Molecular Probes, Eugene, OR, USA) at 37 °C for 1 h. The samples were then analyzed using a flow cytometer.

### Flow cytometric analysis

Flow cytometric analysis was performed as described in the previous study [9]. Briefly, samples were analyzed with flow cytometry using a Gallios™ flow cytometer (Beckman Coulter, Tokyo, Japan) equipped with standard optics. For each cell, forward light scatter, orthogonal light scatter, and Alexa 488 (FL1) were evaluated using the Kaluza® software (Beckman Coulter, Tokyo, Japan). The sperm population of each sample was identified using the side scatter (SSC) and FL1 fluorescence intensities. Debris was gated out by establishing a region around the population of interest, based on FL1/ SSC 2-dimensional histogram. Ten thousand spermatozoa per sample were analyzed. The spermatozoa were labeled with anti-SPMI mouse IgG (SEMG-positive spermatozoa) and positive and negative populations were determined by comparing the population of control staining using mouse IgG isotype control, instead of the antibody (Additional file 1: Figure S1). The proportion of SEMG-positive spermatozoa (SEMG+) was then calculated by dividing the number of labeled spermatozoa by the number of spermatozoa analyzed, and the proportion of SEMG-negative spermatozoa (SEMG−) was determined by subtracting the percentage of SEMG+ from 100. Total SEMG- count was calculated using the proportion of SEMG- and total sperm count.

### Statistical analysis

Statistical analyses comparing mean values of each parameter for patients and control subjects were performed with JMP software (version 10.0.0, SAS Institute Inc., Cary, NC, USA) using Wilcoxon rank sum test. Additionally, the Spearman’s rank correlation coefficient was applied between the results for the proportion of Sg + and the results of standard semen analysis. Statistical analyses comparing differences in the continuous variables were performed with the “R” statistical software system (www.cran.r-project.org) [11] using analysis of variance, followed by Dunnett’s test using the “multcomp” package [12].

## Results

### Parameters from SEMG-labeling analysis and other parameters among infertile male patients

The clinical characteristics and proportions of SEMG+ and SEMG− were examined retrospectively (Table 1). Statistically significant differences were observed between male infertile patients and control subjects regarding all the parameters explored, including the proportion of SEMG+ (71.2 vs. 20.0%, *P* < 0.001). Notably, the number of control subjects was small. This is a reasonable result given that our control subjects were men who were confirmed not to be infertile. The correlation between the proportion of SEMG+ and the results of standard semen analysis among male infertile patients (Table 2) and control subjects (Additional file 2: Table S1) was analyzed. A trend of negative correlation was observed between the proportion of SEMG+ and sperm motility only in male infertile patients, though it was weak (ρ = −0.23, *P* = 0.05), while no significant correlations were observed between the proportion of SEMG+ and sperm concentration, or between SEMG+ and sperm viability. None of the other parameters such as age, serum T level, serum FSH level, and/or serum LH level were significantly correlated with the proportion of SEMG+ in both infertile patients and in control subjects.

**Table 1:** Clinical characteristics and the proportion of SEMG+ and SEMG− in subjects

Characteristics	Patients (*N* = 142)			Control (*N* = 13)		
	Mean ± SD	Median	Min-max	Mean ± SD	Median	Min-max
Age (years)	36.5 ± 6.6	35.5	21–60	30.1 ± 4.1	29.5	25–36
Vol (mL)	4.25 ± 1.73	3.75	1.2–9.3	N/A	N/A	N/A
Conc (×10^6^/mL)	50.6 ± 58.0	33.9	0.0004–400	76.8 ± 7.3	81.5	45.9–167.8
Mot (%)	27.5 ± 19.9	25.75	0–86.1	63.4 ± 2.1	61.05	50.8–86.6
TSC (×10^6^/mL)	208.7 ± 19.8	143.5	0.003–1475	N/A	N/A	N/A
Viability (%)	55.76 ± 19.3	58.7	1.8–87.6	N/A	N/A	N/A
SEMG+ (%)	71.2 ± 20.2	73.5	12.2–99.2	20.0 ± 11.8	18.6	6.1–53.2
SEMG− (%)	28.8 ± 20.2	26.5	0.8–87.8	80.0 ± 11.8	81.4	46.8–93.9

*SEMG+* SEMG-positive spermatozoa, *SEMG−* SEMG-negative spermatozoa; Patients: male infertile patients; Control: normal healthy male subjects with pregnant wives

Mean values of each parameter for patients and control subjects were compared using Wilcoxon rank sum test, which revealed significant differences concerning all parameters compared

Vol (semen volume)

N/A (not applicable)

Conc (sperm concentration)

Mot (total sperm motility)

TSC (total sperm count)

Viability (sperm viability)

**Table 2:** Correlation between the proportion of SEMG+ and standard semen analysis among male infertile patients

Characteristics	Correlation coefficient (ρ)	*P* value
Age (years)	0.00	0.986
Vol (mL)	0.02	0.838
Conc (×10^6^/mL)	−0.12	0.152
Mot (%)	−0.23	0.005
TSC (×10^6^/mL)	−0.14	0.098
Viability (%)	−0.13	0.134
FSH (mIU/mL)	0.13	0.135
LH (mIU /mL)	0.09	0.320
T (ng/dL)	0.00	0.985

ρ: Spearman’s rank correlation coefficient

### Relationship between SEMG-labeling analysis and pregnancy outcomes

Next, we compared the pregnancy and non-pregnancy groups in patients to check if there were any differences in parameters. No parameters, including the proportion of SEMG+/− and total SEMG+/− count, demonstrated significant differences between the two groups (Table 3). Then, we examined whether there were differences in SEMG+/− and/or other parameters between the two groups (pregnancy established with sperm passing through the uterus and oviduct (spontaneous and IUI, in vivo fertilization) versus pregnancy established without sperm passage into the uterus (IVF or ICSI, in vitro fertilization)), since SEMGs are considered to suppress the process of ectopic capacitation in uterus and to be removed from the surface of sperm in oviduct during in vivo fertilization [13–15]. We compared the summed data of spontaneous pregnancy and IUI to that of IVF and ICSI (Table 4). The proportion of SEMG+/− and total SEMG+ count also did not show a difference between these two groups; however, the total SEMG− count in the IVF- or ICSI-pregnancy group tended to be lower than in the spontaneous pregnancy or IUI-pregnancy group (36.7 × 10^6^ vs. 73.4 × 10^6^, *P* = 0.06).

**Table 3:** Comparison between patients whose partners achieved pregnancy and those whose partners did not

Characteristics	Pregnancy (*N* = 36)			Non-pregnancy (*N* = 60)			*P* value
	Mean ± SD	Median	Min-max	Mean ± SD	Median	Min-max	
Age (years)	35 ± 5.8	34.5	21–48	37 ± 6.4	36	26–56	0.24
Vol (mL)	3.9 ± 1.4	3.6	1.2–8	4.0 ± 1.7	3.7	1.2–8.9	0.89
Conc (×10^6^/mL)	49.7 ± 57.0	33.9	0.001–322.8	53.9 ± 68.8	34.8	0.0004–399.9	0.92
Mot (%)	28.8 ± 20.5	26.9	0–86.1	25.6 ± 18.8	24.65	0–66.4	0.43
TSC (×10^6^/mL)	186.3 ± 196.3	145.1	0.005–839.3	279.8 ± 221.0	126.4	0.003–1475	0.89
Viability (%)	57.6 ± 14.8	57.4	21–84.2	55.5 ± 20.1	58.8	1.9–86.6	0.85
FSH (mIU/mL)	4.9 ± 2.7	4.6	1.6–16	5.2 ± 3.8	4.3	1.4–18.8	0.73
LH (mIU/mL)	3.5 ± 1.7	2.8	1.8–7.8	2.9 ± 1.5	2.6	1.3–9.6	0.08
T (ng/dL)	487.7 ± 171.4	480.3	124–1067	463.6 ± 166.6	448	167–834	0.51
SEMG+ (%)	73.9 ± 18.5	73.2	27.6–99	71.9 ± 19.8	73.8	12.2–96.9	0.70
SEMG- (%)	26.1 ± 18.5	26.8	26.8	28.1 ± 19.8	26.2	3.1–87.8	0.70
Total SEMG+ count (×10^6^)	127.7 ± 121.6	101.5	0–548.0	144.8 ± 185.3	77.3	0–1075	0.83
Total SEMG− count (×10^6^)	58.6 ± 96.4	28.7	0–430.2	76.2 ± 143.6	21.0	0.0002–821.5	0.90

Note that only 96 of 142 patients answered the questionnaires regarding pregnancy outcomes and were included in this analysis. Mean values of each parameter for patients and control subjects were compared using Wilcoxon rank sum test

**Table 4:** Comparison within pregnancy groups

Characteristics	SP + IUI (*N* = 20)	IVF+ ICSI (*N* = 16)	*P* value
Age (years)	35 ± 6.1	36 ± 5.5	0.44
Semen volume (mL)	4.0 ± 1.3	3.8 ± 1.4	0.74
Sperm concentration (×10^6^/mL)	56.2 ± 67.0	41.7 ± 42.0	0.34
Sperm motility (%)	31.2 ± 22.3	25.8 ± 18.2	0.67
Sperm viability (%)	59.0 ± 12.9	55.7 ± 17.3	0.91
Total sperm count (×10^6^)	203.6 ± 195.0	163.5 ± 199.4	0.32
Total motile sperm count (×10^6^)	88.4 ± 158.2	65.0 ± 120.1	0.26
Total viable sperm count (×10^6^)	125.7 ± 136.9	103.6 ± 116.2	0.48
SEMG+ (%)	69.9 ± 19.4	78.8 ± 16.6	0.21
Total SEMG+ count (×10^6^)	130.2 ± 103.8	126.9 ± 149.7	0.34
Total SEMG− count (×10^6^)	73.4 ± 114.5	36.7 ± 60.3	0.06

SP + IUI: patients whose wives achieved pregnancy either spontaneously (SP) or through IUI; IVF+ ICSI: patients whose wives achieved pregnancy through IVF or ICSI

## Discussion

Previously, our group reported the association between SEMGs and spermatozoa in infertile men with asthenozoospermia [9]. A significant negative correlation was found between sperm motility and the proportion (*R* = − 0.68) and intensity (*R* = − 0.38) of anti-SPMI labeling, implying that the SPMI, and eventually SEMGs, binding to the sperm surface might account for some disorders in sperm motility that are observed in infertile men with asthenozoospermia.

In this study, we expanded the pool of potential subjects to include infertile patients whose infertility was caused not only by asthenozoospermia but also by oligozoospermia and normozoospermia (cryptogenic male infertility). We further explored the possibility of SEMGs bound to spermatozoa being a predictor of infertility treatment for such a variety of infertile patients in the future. We confirmed a negative correlation between sperm motility and the proportion of SEMG+ in this study, although it was a comparatively weak correlation since the subjects included in this study were not restricted to those with asthenozoospermia (Table 2). For this reason, one might assume that a normal subject bears a lower proportion of SEMG+, since their sperm motility is normal. However, this is not necessarily true since the proportion of SEMG+ is often high, even in an infertile subject whose sperm motility is normal. Therefore, it is possible that the proportion of SEMG+ is an indicator of normal fecundity of the spermatozoa independently of their motility.

The proportion of SEMG+, as well as any other parameters, including sperm motility, did not show a significant difference between the pregnancy and non-pregnancy groups, which indicated that it might not be a general predictor of ability to bear children in an infertile subject. Naturally, the link between SEMGs and the success of pregnancy requires further support. This is because, in addition to factors associated with the male, factors associated with the female, the fertilized egg, and the early development of the embryo are all involved in the success of pregnancy. To conduct an analysis while considering these confounding factors, it is necessary to further investigate increasing the number of specimens.

Next, based on the hypothesis that the condition of SEMGs bound to spermatozoa differs between pregnancy achieved through IUI or spontaneous pregnancy and that achieved through IVF or ICSI, we focused on the result of 36 cases in which conception was achieved. When the summed data of spontaneous pregnancy and IUI was compared to the summed data of IVF and ICSI, the total SEMG− count was apparently higher in the former. Such a tendency was not observed in any of the other parameters, including the proportion of SEMG+. Although the precise molecular mechanism underlying the controlling fertilizing capacity of sperm in vivo have not been revealed, the role of SEMGs on sperm were well studied and proved to control motility and capacitation [8, 16–18]. Moreover, according to our analysis in knockout mice, seminal vesicle secretion 2 (SVS2), an orthologue of SEMGs, is essential for in vivo fertilization [13]. SVS2 is thought to be a protector against spermicidal agents in the uterus, and to be removed from the sperm’s surface after passing through the isthmus [14]. Coincidently, the removal of SVS2 induces the decrease of cholesterol from the sperm membrane, thereby resulting in the ability of sperm to fertilize the egg [15]. The data in this study indicate that a patient with a larger number of spermatozoa with removed SEMGs can be involved in a successful pregnancy via in vivo fertilization. The removal of SEMGs from the surface of spermatozoon under normal conditions allows for sperm to fertilize an egg. Furthermore, the number of such spermatozoa is important for in vivo fertilization.

While results from this study provide a minimum-requirement analysis of the SEMGs bound to spermatozoa and conventional semen analysis focused on pregnancy outcomes, this study also provides the need for further large-scale studies to establish diagnostic certainty. In addition to this study having a small patient population, this study has a limitation that the characteristics of the female partners, such as age and oocyte quality, are not considered. Pregnancy outcomes of a couple are usually strongly affected by the condition of the female partner. Thus, it is possible that the influence of male factors on pregnancy outcomes is importantly obscured under such uncontrolled conditions of the female. Surprisingly, SEMGs were found to be potential biomarkers that can guide a couple to select a suitable method of assisted reproductive technology. They were also found to be particularly valuable for the decision to use IVF or ICSI rather than IUI. Usually, natural pregnancy or IUI using the timing method are used in infertile couples with no particular female factors when the male partner is normozoospermic or mildly oligozoospermic or asthenozoospermic. According to the findings of this study, it is likely that even such a couple should consider early step-up using the data of total SEMG− count. More data must be collected to evidence this hypothesis and to determine any cut-off value. In the future, SEMGs bound to sperm may be used to determine whether IUI, IVF, or ICSI treatment should be administered promptly, without waiting for spontaneous pregnancy, in case of couples who desire to have their own child.

## Conclusions

Regarding the partner of patient pregnancy subgroup, the total SEMG− count tended to be high in patients whose partners had spontaneous pregnancy or who underwent IUI treatment, compared with partners of patients who used other treatments. This result suggests that SEMGs inhibit ectopic capacitation before sperm reach the fertilization site and that the number of total SEMG-unbound sperm is a parameter directly linked to the possibility of in vivo fertilization. It is possible that total SEMG− count might be a tool for determining whether IUI, spontaneous conception, IVF, or ICSI should be used for the partners’ treatment of ART. It is necessary to perform further large-scale studies to establish the diagnostic certainty of SEMGs bound to spermatozoa.

## Additional files

Additional file 1: Figure S1. Representative histograms obtained using flow cytometry. Subpopulation of SEMG+ was determind by compearing to unstained sperm (mIgG isotype control). (PDF 1133 kb)

Additional file 2: Table S1. Correlation between the proportion of SEMG+ and the result of standard semen analysis among control subjects (*N* = 13). (DOCX 10 kb)

## Abbreviations

ART: Assisted reproductive techniques
CV: Coefficient of variation
FSH: Follicle-stimulating hormone
HBS: HEPES-buffered saline
ICSI: Intra cytoplasmic sperm injection
IgG: Immunoglobulin G
IUI: Intra-uterine insemination
IVF: In vitro fertilization
LH: Luteinizing hormone
PSA: Prostate-specific antigen
SEMG-: SEMG-unbound spermatozoa
SEMG+: SEMG-bound spermatozoa
SEMGs: Semenogelins
SPMI: Seminal plasma motility inhibitor
SSC: Side scattering
T: Testosterone

## References

[1] Jungwirth A, Diemer T, Dohle GR, Giwercman A, Kopa Z, Krausz C, Tournaye H. Guidelines for Male Infertility. In: European Association of Urology 2012. https://uroweb.org/wp-content/uploads/17-Male-Infertility_LR1.pdf. Accessed 23 Mar 2017. 10.1016/j.eururo.2012.04.048 22591628

[2] Esteves SC, Sharma RK, Gosalvez J, Agarwal A (2014). A translational medicine appraisal of specialized andrology testing in unexplained male infertility. Int Urol Nephrol.

[3] Guzick DS, Overstreet JW, Factor-Litvak P, Brazil CK, Nakajima ST, Coutifaris C (2001). The National Cooperative Reproductive Medicine Network. Sperm morphology, motility, and concentration in fertile and infertile men. N Engl J Med.

[4] Hamada A, Esteves SC, Nizza M, Agarwal A (2012). Unexplained male infertility: diagnosis and management. Int Braz J Urol.

[5] Lilja H, Abrahamsson PA, Lundwall A (1989). Semenogelin, the predominant protein in human semen. Primary structure and identification of closely related proteins in the male accessory sex glands and on the spermatozoa. J Biol Chem.

[6] Yoshida K, Iwamoto T, Yoshida M, Lejeune T, Delvaux P (2010). Effects of the seminal plasma proteins semenogelin (SEMG)/seminal vesicle secretion 2 (SVS2) on sperm fertility. Human Spermatozoa: Maturation, Capacitation and Abnormalities.

[7] Robert M, Gagnon C (1999). Semenogelin I: a coagulum forming, multifunctional seminal vesicle protein. Cell Mol Life Sci.

[8] Esfandiari N, de Lamirande E, Guktulket A, San Gabriel MC, Nazemian Z, Burjaq H (2014). Seminal hyperviscosity is not associated with semenogelin degradation or sperm deoxyribonucleic acid damage: a prospective study of infertile couples. Fertil Steril.

[9] Terai K, Yoshida K, Yoshiike M, Fujime M, Iwamoto T (2010). Association of seminal plasma motility inhibitors/semenogelins with sperm in asthenozoospermia-infertile men. Urol Int.

[10] Yoshida K, Yamasaki T, Yoshiike M, Takano S, Sato I, Iwamoto T (2003). Quantification of seminal plasma motility inhibitor/semenogelin in human seminal plasma. J Androl.

[11] The R Project for Statistical Computing: The R Foundation. http://www.R-project.org, 2015. Accessed 23 Mar 2017.

[12] Hothorn T, Bretz F, Westfall P (2008). Simultaneous: inference in general parametric models. Biom J.

[13] Kawano N, Araki N, Yoshida K, Hibino T, Ohnami N, Makino M (2014). Seminal vesicle protein SVS2 is required for sperm survival in the uterus. Proc Natl Acad Sci U S A.

[14] Kawano N, Yoshida M (2007). Semen-coagulating protein, SVS2, in mouse seminal plasma controls sperm fertility. Biol Reprod.

[15] Araki N, Trencsényi G, Krasznai ZT, Nizsalóczki E, Sakamoto A, Kawano N (2015). Seminal vesicle secretion 2 acts as a protectant of sperm sterols and prevents ectopic sperm capacitation in mice. Biol Reprod.

[16] Yoshida K, Krasznai ZT, Krasznai Z, Yoshiike M, Kawano N, Yoshida M (2009). Functional implications of membrane modification with semenogelins for inhibition of sperm motility in humans. Cell Motil Cytoskeleton.

[17] Yoshida K, Kawano N, Yoshiike M, Yoshida M, Iwamoto T, Morisawa M (2008). Physiological roles of semenogelin I and zinc in sperm motility and semen coagulation on ejaculation in humans. Mol Hum Reprod.

[18] de Lamirande E, Yoshida K, Yoshiike TM, Iwamoto T, Gagnon C (2001). Semenogelin, the main protein of semen coagulum, inhibits human sperm capacitation by interfering with the superoxide anion generated during this process. J Androl.

