# Supplementary figures and images for: Relationship between Semenogelins bound to human sperm and other semen parameters and pregnancy outcomes
Source: Basic Clin Androl. 2017 Aug 8;27:15. doi: 10.1186/s12610-017-0059-6 (PMC5547539; doi:10.1186/s12610-017-0059-6)

Typical case 1(AIH): proportion of SEMG+ was 49.04%

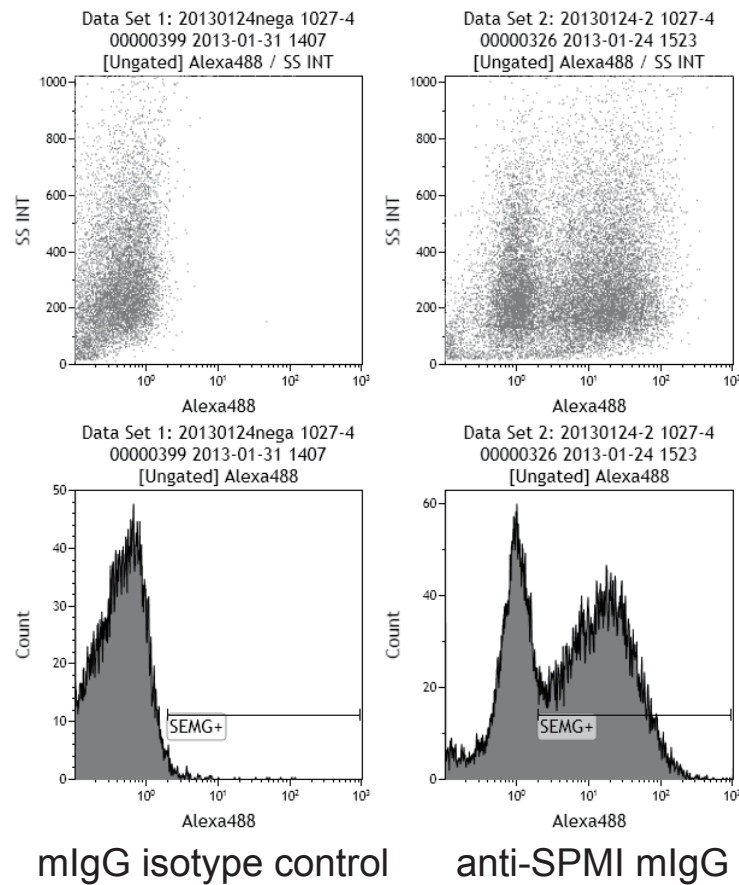

Typical case 2(IVF): proportion of SEMG+ was 93.97%

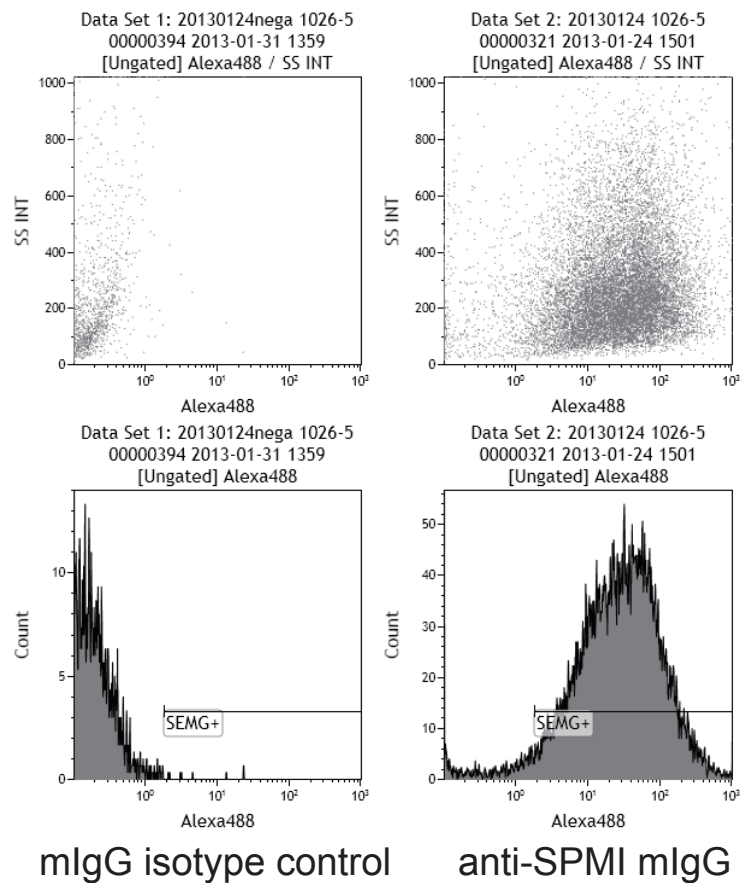

Supplement: Supplementary file 1 — Representative histograms obtained using flow cytometry. Subpopulation of SEMG+ was determind by compearing to unstained sperm (mIgG isotype control). (PDF 1133 kb) [file 12610_2017_59_MOESM1_ESM.pdf]
