# Supplementary material for: Relationship between Semenogelins bound to human sperm and other semen parameters and pregnancy outcomes
Source: Basic Clin Androl. 2017 Aug 8;27:15. doi: 10.1186/s12610-017-0059-6 (PMC5547539; doi:10.1186/s12610-017-0059-6)
Supplement: Supplementary file 2 — Correlation between the proportion of SEMG+ and the result of standard semen analysis among control subjects (N = 13). (DOCX 10 kb) [file 12610_2017_59_MOESM2_ESM.docx]

Table S1. Correlation between the proportion of SEMG＋ and the result of standard semen analysis among control subjects (N =13)

Characteristics Correlation coefficient (ρ) P value

Age (years) 0.07 0.835

Sperm concentration (×10^6^/mL) 0.104 0.734

Sperm motility (%) -0.27 0.374

ρ: Spearman’s rank correlation coefficient.
